# Supplementary material for: AB186 Inhibits Migration of Triple-Negative Breast Cancer Cells and Interacts with α-Tubulin
Source: Int J Mol Sci. 2022 Jun 20;23(12):6859. doi: 10.3390/ijms23126859 (PMC9225035; doi:10.3390/ijms23126859)
Supplement: Supplementary file 1 [file ijms-23-06859-s001.zip › ijms-1764232-supplementary.pdf]

Supplementary data-S1

Synthesis of tagged derivatives of AB186

1. General methods

Solvents and liquid reagents were purified and dried according to recommended procedures. Chemical reagents were purchased from Merck, Fisher Scientific or TCI Europe and were used as received. Analytical thin-layer chromatography was performed with silica gel 60 F254, 0.25 mm pre-coated TLC plates (Merck). Compounds were visualized using UV light (254 nm) and a solution of cerium sulfate tetrahydrate and phosphomolybdic acid in 10% aqueous sulfuric acid as developing agent. Column chromatographies were performed using a Grace Reveleris® apparatus using 40 µM silica gel. NMR spectrum was recorded at 303K on a Bruker DRX400 (400 MHz for ^{1}H and 100.6 MHz for ^{13}C) spectrometer. The chemical shifts are reported in ppm (δ) relative to residual solvent peak. Mass spectra (MS) were recorded on a micrOTOFq (Bruker) ESI/QqTOF spectrometer. Melting points (M.p.) were determined with a Kofler bench and are uncorrected. FTIR spectra were recorded on a Shimadzu IRAffinity-1 apparatus equipped with an ATR PIKE diamond gladiATR.

2. Synthesis of fluorescent derivative of AB186 (ABRhod)

(*Z*)-*N*-(9-(2-carboxy-4-(3-(6-(2-((4-((2,4-dioxothiazolidin-5-ylidene)methyl)phenoxy)methyl)-2,5,7,8-tetramethylchroman-6-yl)hexyl)thioureido)phenyl)-6-(diethylamino)-3*H*-xanthen-3-ylidene)-*N*-ethylethanaminium (mixture of regioisomers).

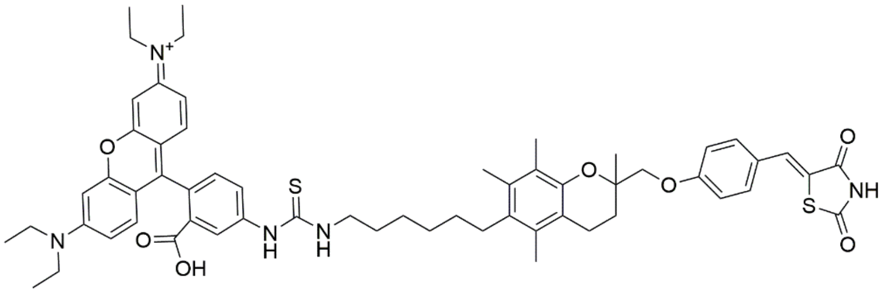

To a solution of AB186 (23 mg, 36 µmol) in dry EtOH (1ml) was added triethylamine (2.2 µL, 54.2 µmol). The resulting precipitate was dissolved by the addition of CH_2Cl_2 (0.2 mL). To this solution was added RhodamineB isothiocyanate (19.4 mg, 36.1 µmol) and the reaction was stirred at room temperature protected from the light for 4 days until the total consumption of the starting material. The solvents were evaporated under vacuum and the crude was purified by column chromatography (eluent: CH_2Cl_2/MeOH, 9/1) to obtain ABRhod (21 mg, 20.5 µmol, 57% yield) as a dark red solid. M.p. 161-163 °C. IR (ATR): ν (cm^{-1}): 2920, 2853, 1682, 1585, 1337, 1246, 1177. ESI-MS (pos. mode): calculated m/z = 1022.4555. Found: 1022.4584 $[M]^{+}$.

3. Synthesis of biotinoyl AB186 (ABBiot)

N-(6-(2-((4-((*Z*)-(2,4-dioxothiazolidin-5-ylidene)methyl)phenoxy)methyl)-2,5,7,8-tetramethylchroman-6-yl)hexyl)-5-((3a*S*,4*S*,6a*R*)-2-oxohexahydro-1*H*-thieno[3,4-*d*]imidazol-4-yl)pentanamide

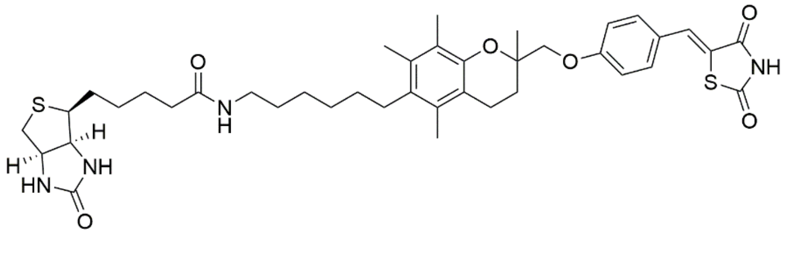

To a stirred solution of biotin (38.3 mg, 157 µmol) in DMF (4 mL) were added triethylamine (56 µL, 403 µmol) and dichloromethane (2 mL). The solution was then cooled at 0°C and *iso*butyl chloroformate (20.4 µL, 157 µmol) was added. After 45 min under stirring at this temperature, a solution of AB186 trifluoroacetate (100 mg, 157 µmol) in DMF (2 mL) was added and the mixture was stirred at room temperature for 12 hours. The solution was then diluted with EtOAc (100 mL), the organic layer was washed with 5% aqueous citric acid solution (2 × 30 mL), 5 % aqueous $NaHCO_3$ solution (2 × 30 mL) and brine (2 × 30 mL). The organic layer was then dried ($MgSO_4$) and the solvents were evaporated under reduced pressure to give a yellow oil which was purified upon flash column chromatography (eluent: MeOH/CH_2Cl_2 0/100 to 7/93 in 25 minutes) to give 46 mg of a slightly yellow powder (61 µmol, 39 %). M.p. 149-151°C. IR (ATR) ν (cm^{-1}): 3424, 2920, 2853, 1738, 1682, 1597, 1510, 1250. ^1H NMR (DMSO-d_6, 400 MHz): 1.22-1.66 (m, 14H, 7 × linker-CH_2), 1.31 (s, 3H, CH_3), 1.78-1.88 (m, 1H, chromane 3-CH_aH_b), 1.95 (s, 3H, CH_3), 1.96-2.06 (m, 1H, chromane 3-CH_aCH_b), 2.04 (t, *J* = 7.3 Hz, 2H, linker-CH_2), 2.09 (s, 6H, 2 × CH_3), 2.46-2.63 (m, 6H, 2 CH_2 + 2 CH), 2.61 (t, *J* = 7.3 Hz, 2H, chromane 4-CH_2), 3.03 (q, *J* = 6.3 Hz, 2H, CH_2), 4.04-4.12 (m, 2H, chromane 2-CH_2-O), 4.06-4.13 (m, 1H, biotin CH-NH), 4.24-4.31 (m, 1H, biotin CH-NH), 6.34 (s, 1H, biotin NH), 6.41 (s, 1H, biotin NH), 7.13 (d, *J* = 8.9 Hz, 2H, CHAr), 7.52 (d, *J* = 8.9 Hz, 2H, CHAr), 7.70 (s, 1H, CH=C), 7.73 (t, *J* = 5.6 Hz, 1H, NH). ^{13}C NMR (DMSO-d_6, 100.6 MHz): 11.9 (CH_3), 14.1 (CH_2), 14.6 (CH_3), 15.5 (CH_3), 21.6 (CH_3), 22.3 (CH_2), 25.4 (CH_2), 26.2 (CH_2), 28.1 (biotin CH_2), 28.2 (biotin CH_2), 29.2 (chromane 3-CH_2), 29.2 (biotin CH_2), 29.4 (linker CH_2), 29.6 (CH_2), 35.2 (biotin CH_2), 38.3 (CH_2), 40.2 (CH_2-S), 55.4 (CH-S), 59.0, 59.2 (CH-NH), 59.8, 61.0 (CH-NH), 72.6 (CH_2-O), 73.9 (chromane 2-C), 115.6 (CHAr), 116.5, 120.9, 125.9, 131.2, 131.0, 131.9 (CHAr), 132.5 (CH=C), 148.4, 160.3, 162.7, 168.6 171.8. ESI-MS (pos. mode): calculated m/z = 771.3220. Found: 771.3226 $[M+Na]^+$.

Supplementary data-S2

Materials

Mercapto-1-undecanol (MUOH), mercapto-1-hexadecanoic acid (MHA), N-hydroxysulfosuccinimide (S-NHS), tris(2-carboxyethyl)phosphine (TCEP), acetonitrile, NH_4HCO_3, and rat serum albumin (RSA), N-(3-Dimethylaminopropyl)-N-ethyl-carbodiimide (EDC), and triethylamine (TEA), ethanolamine (HCl, pH 8.5, 1 M), Octyl-Glucopyranoside (OG) were purchased from Merck (Darmstadt, Germany). Digest from bovine serum albumin (BSA) and α-cyano-4-hydroxycinnamic acid (HCCA) were purchased from Bruker Daltonics (Bremen, Germany). Absolute Ethanol is provided by VWR International (Fontenay sous Bois, France). The running buffer was phosphate buffered saline (PBS), 10 mM at pH 7.4 with NaCl (138 mM), KCl (2.7 mM) and Tween 20 (0.05%). For protein digestion, we used trypsin enzyme (Gold Mass Spectrometry Grade from Promega) in sodium acetate buffer at 10 mM. All of the buffers were prepared using ultrapure water (18 MΩ/cm resistance, Purelab prima from Elga LabWater, Antony, France).

SPRi Analysis

Homemade chips were chemically functionalized with a self-assembled monolayer composed of a mixture of MUOH and MHA at 1 mM (90/10 by mole) in absolute ethanol. The sensor chips were cleaned with absolute ethanol (Carlo Erba: Val de Reuil, France) then treated overnight and rinsed with ultra-pure ethanol and water (Elga LabWater, Antony, France). Afterwards, in the SPRi-Plex apparatus (HORIBA Scientific, Palaiseau, France), carboxyl groups of MHA are activated for 10 minutes at 20 µL/min by one injection of a solution of 100 mM S-NHS and of 400 mM EDC. Solution of AB186 (50 mM DMSO) was made in 5mM in absolute ethanol and triethylamine. Final solution of AB186 was obtained in PBS (pH 7.4 with NaCl 138 mM, KCl 2.7 mM) to a final concentration of 50 µM. 200 µL of AB186 solution was injected at 20 µL/min following by a washing step by OG (40mM) was performed during 1 min at 50 µl/min. Deactivation of free S-NHS sites on the chip was performed in one injection of ethanolamine (1M in HCl) during 14 minutes at 10 µL/min.

Prior injection of cytoplasmic proteins (130 µg/mL), a filtration step was performed by microSPIN COSTAR (0.2 µm) at 4 ℃, 10 000 rpm and during 7 min. Injection of the sample (5 µg/ml) was performed in PBS at 25 ℃ at a flow rate of 20 µL/min for 10 minutes, repeated twice following by a washing step of OG 40mM. At the end of the interaction, the chip was removed from the apparatus and rinsed with ultra-pure water to be analyzed by mass spectrometry.

On-Chip Reduction, Tryptic Digestion and On-Chip MS analysis

The treatments of the captured proteins were performed directly on chip using ImagePrep Station (Bruker Daltonics, Bremen, Germany) and included the following steps: reduction step (TCEP 10 mM in 0.1 M NH_4HCO_3, Sigma Aldrich) followed by a tryptic digestion step (10–40 ng/µL) both at 37 ℃ and the matrix deposition step (HCCA (1 mg/mL) in 50/50 v/v water/acetonitrile with 0.25%TFA) at room temperature. The biochip was placed in the UltrafleXtreme MALDI-TOF/TOF (Bruker Daltonics, Bremen, Germany) using a prototype adapter target designed for biochips. The UltrafleXtreme MALDI was equipped with a smartbeam-II laser (1,000 Hz repetition rate), which enables ultra-high data acquisition speed in both MS and MS/MS. The raw MS data were processed in Proteome Discoverer software. A local Mascot server (Mascot version 2.2.01; Matrix Science, Boston, MA, USA) and the Swiss-Prot TrEMBL database was used for protein identification based on MS or MS/MS spectra.
